# The serum metabolomic profile of a distinct, inflammatory subtype of acute psychosis

**DOI:** 10.1038/s41380-022-01784-4

**Published:** 2022-09-21

**Authors:** Belinda Lennox, Wenzheng Xiong, Patrick Waters, Alasdair Coles, Peter B. Jones, Tianrong Yeo, Jeanne Tan May May, Ksenija Yeeles, Daniel Anthony, Fay Probert

**Affiliations:** 1 Department of Psychiatry, University of Oxford and Oxford Health NHS Foundation Trust, Oxford, UK; 2 Department of Pharmacology, University of Oxford, Oxford, UK; 3 Department of Chemistry, University of Oxford, Oxford, UK; 4 Nuffield Department of Clinical Neurosciences, University of Oxford, Oxford, UK; 5 Department of Clinical Neurosciences, University of Cambridge, Cambridge, UK; 6 Department of Psychiatry, University of Cambridge, Cambridge, UK; 7 Department of Neurology, National Neuroscience Institute, Singapore, Singapore; 8 Duke-NUS Medical School, Singapore, Singapore

**Keywords:** Diagnostic markers, Neuroscience

## Abstract

A range of studies suggest that a proportion of psychosis may have an autoimmune basis, but this has not translated through into clinical practice—there is no biochemical test able to accurately identify psychosis resulting from an underlying inflammatory cause. Such a test would be an important step towards identifying who might require different treatments and have the potential to improve outcomes for patients. To identify novel subgroups within patients with acute psychosis we measured the serum nuclear magnetic resonance (NMR) metabolite profiles of 75 patients who had identified antibodies (anti-glycine receptor [GlyR], voltage-gated potassium channel [VGKC], Contactin-associated protein-like 2 [CASPR2], leucine-rich glioma inactivated 1 [LGI1], N-methyl-D-aspartate receptor [NMDAR] antibody) and 70 antibody negative patients matched for age, gender, and ethnicity. Clinical symptoms were assessed using the positive and negative syndrome scale (PANSS). Unsupervised principal component analysis identified two distinct biochemical signatures within the cohort. Orthogonal partial least squared discriminatory analysis revealed that the serum metabolomes of NMDAR, LGI1, and CASPR2 antibody psychosis patients were indistinct from the antibody negative control group while VGKC and GlyR antibody patients had significantly decreased lipoprotein fatty acids and increased amino acid concentrations. Furthermore, these patients had more severe presentation with higher PANSS scores than either the antibody negative controls or the NMDAR, LGI1, and CASPR2 antibody groups. These results suggest that a proportion of patients with acute psychosis have a distinct clinical and biochemical phenotype that may indicate an inflammatory subtype.

## Introduction

There are many lines of evidence to indicate that a proportion of psychosis is linked to increased inflammation and may have an autoimmune basis. Much of this evidence is indirect, such as the association between schizophrenia and genes important for the adaptive immune system [1, 2], and the higher rate of other autoimmune disorders in those with schizophrenia than other people [3, 4]. A more direct clue has come from the identification of antibodies against neuronal cell surface targets in some patients with psychosis [5]. These antibodies, when found in patients with other clinical presentations, in particular autoimmune encephalitis, are considered causative, and removal of the antibodies treats the associated clinical presentation [6–8].

Nuclear magnetic resonance (NMR) metabolomics analysis of biofluid samples is a rapidly growing field which has been shown to identify systemic inflammation and antibody-mediated pathology in a range of diseases [9–11]. Recent studies suggest that the serum metabolome may provide a more sensitive measure of low-grade inflammation than current, clinically used, measures [12, 13]. In addition, the application of unsupervised cluster analysis to metabolomics data can reveal novel metabolic phenotypes within patient cohorts [9, 14], inspection of the small molecules responsible for this clustering can then reveal distinct biochemical pathway changes in these groups. Here we apply NMR serum metabolomics analysis coupled with unsupervised pattern recognition methods to identify novel subgroups within a cohort of psychosis patients and relate the identified metabolic phenotypes to measures of disease severity at presentation, using the positive and negative syndrome scale (PANSS), and the presence of a range of neuronal cell surface antibodies in the serum of these patients.

We have been screening patients with psychosis for neuronal cell surface antibodies for the past nine years. Over this time the range of antibodies that has been recognised has expanded, and the evidence around the pathogenicity of some of these antibodies has grown. For instance, pre-clinical studies support the pathogenicity of the N-methyl-D-aspartate receptor (NMDAR) antibody in patients with autoimmune encephalitis [15], and the nature of the clinical syndrome is well characterised [6, 16]. The clinical relevance of these antibodies in those with psychosis is less certain, even though there are clear overlaps in terms of symptoms seen [17]. Studies that have directly examined NMDAR antibodies from patients with psychosis also demonstrate their functional effects on synaptic function [18], and small case series of patients with psychosis and NMDAR antibodies demonstrate a comparable treatment response to immunotherapy [19, 20]. By contrast, the evidence around the pathogenicity of antibodies targeting Voltage-Gated Potassium Channel (VGKC) is controversial across all clinical presentations. This was the first neuronal cell surface antibody to be described in association with encephalitis in 2001 [21]. However, more recent studies have specified the particular targets as being components of the VGKC – Leucine-rich glioma-inactivated 1 (LGI1) and Contactin Associated Protein-like 2 (CASPR2) [22], such that the value of testing for generic VGKC antibodies, using radioimmunoassay is now questioned in encephalitis [23], and the clinical recommendation is to only test for LGI1 and CASPR2 [24]. However, the utility of VGKC antibody testing in neurological diseases other than encephalitis remains to be seen. Indeed, other studies indicate that VGKC antibody assays do still have value, indicating an immunotherapy responsive illness in children for instance [25]. There are further antibodies where clinical relevance in CNS disorders is more unclear, such as Glycine receptor (GlyR) antibodies, more associated with progressive encephalomyelitis with rigidity and myoclonus [26], and others that are so rare that there is little to guide clinical practice (GABA-A antibodies) [27].

In order to explore further understand the distinct metabolic phenotypes identified in this psychosis cohort and investigate the possible relevance of these antibodies that we have identified in patients with psychosis, we included a cohort of antibody-positive patients, age and gender matched to patients negative for all antibodies tested, in our cohort. We had the hypothesis that those with defined neuronal cell surface antibodies would have a different metabolomic profile than those without. Furthermore, we explored whether those patients with an inflammatory metabolomic profile had a distinguishing clinical profile, to enable clinicians to detect patients who may need treating in a different way.

## Materials/Subjects and Methods

### Study participants

Serum samples were collected from 1574 patients with acute psychosis across England as part of the Medical Research Council (MRC) Prevalence of Pathogenic Antibodies in Psychosis study (PPiP1 (2013–2014) and PPiP2 2015–2018). The inclusion criteria for the study were ages 16–35, first episode of psychotic illness, antipsychotic medication less than 6 weeks, and at least one moderate or more severe symptom of psychosis. These criteria were then broadened in 2016 to widen the age range 16–70 and extend the length of illness to 24 months and included those at relapse as well as first episode.

Following informed consent, a serum sample was collected, alongside demographic details and a clinician rating of severity of selected positive and negative syndrome scale (PANSS) items at the same time point (P1 Delusions, P2 Conceptual disorganisation, P3 Hallucinatory behaviour, N1 Blunted Affect, N4 Passive/apathetic social withdrawal, N6 Lack of spontaneity and flow of conversation, G5 Mannerisms and posturing and G9 Unusual thought content) [28]. The items mentioned above were rated on a 7-point scale: 1 = absent, 2 = minimal, 3 = mild, 4 = moderate, 5 = moderate severe, 6 = severe, and 7 = extreme [28]. Clinicians were asked to only rate the symptoms that were moderate or more severe (≥4). Absent-mild symptoms did not have ratings by clinicians, and their item score was considered as 1 during statistical analyses. Total and subscales’ scores were calculated with the eight items mentioned above.

For the participants in PPiP1 a full PANSS rating was obtained. This therefore includes ratings of specific delusions of suspiciousness or grandiosity (P6 and P7) that were not included in the brief PANSS ratings. We therefore allocated a single P1 rating for these participants taking the highest scoring item from P1, P6 and P7 as the rating for P1 for these participants.

A total of 145 patients with sufficient serum sample volume for both antibody testing and metabolomics analysis were included in this study; 75 patients who were antibody positive along with 70 antibody negative controls matched for age, gender, and ethnicity, and illness course. Two patients were positive for more than one Ab and so were excluded from model training.

### Standard protocol approvals, registrations, and patient consents

Written informed consent was obtained from all patients according to the Declaration of Helsinki. Ethical approval was obtained by the local research ethics committee (12/EE/0307 PID 97740).

### Serum collection

Serum was collected in a serum separator tube, supplied by the study team, and posted to Oxford. Samples were allowed to clot at room temperature before being centrifuged at 1300 x *g* for 10 min at room temperature. The serum supernatant was immediately stored at −80 °C.

### Antibody testing

Antibody testing for NMDAR, GlyR, GABAA, LGI1, CASPR2 IgG antibodies was undertaken in Nuffield Department Clinical Neuroscience, according to published methods [6, 22, 29]. Voltage Gated Potassium Channel (VGKC) Antibodies were measured by radioimmunoassay in a National Health Service (NHS) Clinical laboratory (Oxford University Hospitals NHS Trust). A threshold of 100 pM was taken as the cut-off for a positive result. All samples collected were tested for all antibodies.

### Nuclear magnetic resonance (NMR) sample preparation for metabolomics analysis

Serum samples were thawed at room temperature and centrifuged at 100,000 × *g* for 30 min at 4 °C. 150 µL of supernatant was then diluted with 450 μL of 75 mM sodium phosphate buffer prepared in D_2_O (pH 7.4). Samples were briefly centrifuged at 3000 × *g* for 5 min before transferring to a 5-mm NMR tube.

All NMR spectra were acquired at 310 K using a 700-MHz Bruker AVIII spectrometer operating at 16.4 T equipped with a ^1^H [^13^C/^15^N] TCI cryoprobe (Department of Chemistry, University of Oxford). The Carr-Purcell-Meiboom-Gill (CPMG) pulse sequence was used to suppress large protein resonance. Quality control samples were randomly spread throughout the acquisition to ensure reproducibility. All spectra were preprocessed in Topspin 2.1 (Bruker, Germany); multiplied by a 1D exponential corresponding to a 0.3 Hz line broadening, and zero filled by a factor of 2. All spectra were baseline corrected with a fifth-degree polynomial and referenced to the lactate doublet at 1.33 ppm. Processed spectra were exported to ACD/Labs Spectrus Processor Academic Edition 12.01 (Advanced Chemistry Development, Inc., Toronto, Canada), whereby regions of the spectra between 0.80–8.47, excluding the water resonance (4.13-5.22 ppm), were split into 0.02-ppm-wide bins which were integrated and exported as a.csv file for statistical analysis. Metabolite assignment was performed by referencing to literature values, the Human Metabolome Database [30], and via 2D total correlation spectroscopy (TOCSY) experiments. NMR-detectable serum/plasma metabolites have been previously reported [31–33]. While all metabolite resonances were included in our analysis, most abundant metabolites detectable in serum NMR spectra included 3-hydroxybutyrate, acetoacetate, alanine, arginine, citrate, creatine, creatinine, formate, glucose, glutamate, glutamine, histidine, lactate, lipoproteins (high-density lipoproteins [HDL], low-density lipoproteins [LDL], very low-density lipoproteins [VLDL], and chylomicron), lysine, myo-inositol, N-acetyl glycoproteins (GlycA, GlycB), phenylalanine, proline, scyllo-inositol, threonine, tyrosine, urea, and valine.

### Statistical analysis

Analysis was performed in R software 3.4.3 (R foundation for statistical computing, Vienna, Austria) and GraphPad Prism 9.0.0. Mann-Whitney U test was used for non-normal continuous variables in two-group comparisons. Ordinary one-way ANOVA (with post hoc Tukey’s multiple comparisons test) was used for continuous variables in comparisons of more than two groups. When unequal variance present among groups, Brown-Forsythe and Welch ANOVA tests (with Dunnett’s T3 multiple comparisons test) were used instead. For variables with non-normal distribution or limited group sample size, Kruskal-Wallis test with Dunn’s multiple comparisons test was used in comparisons of more than two groups. Fisher’s exact tests were used for categorical variables as appropriate, while a Bonferroni correction was applied throughout to account for multiple comparisons. Two-tailed *p*-values ≤ 0.05 were considered statistically significant.

A power calculation (PPCA model using the R package MetSizeR) [34–36] confirmed that a sample size of 12 per group (total 24) is sufficient to achieve an FDR cut-off of 0.05 assuming that 20% of the spectral bins measured significantly differ between groups. Thus, the 19 samples collected within the smallest group (VGKC/GlyR) are sufficient to identify metabolite changes while also allowing a ‘test’ set to be removed.

Multivariate analysis of metabolomics data was carried out using principal component analysis (PCA), an unsupervised analysis showing spontaneous separation of groups, and orthogonal partial least squares discriminant analysis (OPLS-DA), a supervised method to identify significant metabolite changes between groups. All multivariate analysis was using in-house R scripts and the ropls package [37]. OPLS-DA models were validated on independent test data (10%) using an external 10-fold cross-validation strategy with repetition coupled with permutation testing as previously described [12]. A detailed explanation of the cross-validation strategy is included in the supplementary material (Supplementary Fig. 1). All model performance metrics (accuracy, sensitivity, and specificity) are considered significant if they are greater than the corresponding metrics from the random null distribution, determined by the Kolmogorov–Smirnov. Discriminatory variables were identified by calculating the average of the variable importance in projection (VIP) scores of the ensemble of models, which indicated the contribution of a variable to the model. An inflection point was picked manually in the curve of VIP scores and was used as a cut-off for picking discriminatory metabolites in multivariate. Fold changes of discriminatory variables were then calculated and one-way ANOVA with Tukey’s post-hoc corrections were conducted to determine group differences. Two-tailed *p*-values ≤ 0.05 were considered statistically significant in univariate.

## Results and Discussion

### Patient demographics and antibody status

The most prevalent serum antibodies detected in those with antibodies were NMDAR antibodies (47%, *n* = 35), followed by VGKC antibodies (19%, *n* = 13). Two double-positive patients were identified, one positive for both CASPR2 and VGKC antibodies and another positive for both CASPR2 and NMDAR antibodies and so these were excluded from model training. The groups were well matched for age, gender, ethnicity, episode type, and illness duration. The VGKC antibody-positive group had higher PANSS total compared to the Control group (*n* = 70, *p* < 0.01, Kruskal-Wallis test), LGI1 antibody positive group (*n* = 9, *p* < 0.05, Kruskal-Wallis test) and NMDAR antibody positive group (*n* = 35, *p* < 0.05, Kruskal-Wallis test) (Table 1).

**Table 1:** Patient information, grouped by different neuronal cell surface antibodies (*n* = 143).

	Ab negative (Ctrl)	Ab positive	GlyR-Ab	VGKC-Ab	CASPR2-Ab	LGI1-Ab	NMDAR-Ab
**Number of patients**	70	73	7	13	9	9	35
**Age (years), median (IQR)**	27 (15)	25 (13)	24 (10)	23 (8)	24 (11.5)	25 (15)	32 (18)
**Gender, Female, no. (%)**	31 (44.3)	30 (41.1)	4 (57.1)	5 (38.5)	3 (33.3)	1 (11.1)	17 (48.6)
**Ethnicity (%)**							
White	71.4	67.1	100	61.5	55.6	77.8	62.9
Asian	12.9	12.3	0	0	22.2	11.1	17.1
Black (African/Caribbean)	7.1	9.5	0	23.1	11.1	11.1	5.7
Mixed	4.3	6.8	0	7.7	11.1	0	8.6
Other	4.3	0	0	0	0	0	0
Unknown	0	4.1	0	7.7	0	0	5.7
**Disease duration (duration from the episode date to the consent date, days), median (IQR)**	86 (258.8)	103 (244.5)	267 (399)	37 (23)	230 (317)	132 (404)	113 (229)
Unknown, no.	0	4	2	1	0	1	0
**Episode type, no. (%)**							
First episode	45 (64.3)	50 (68.5)	7 (100)	13 (100) (adjusted *p* value = 0.51)	6 (66.7)	7 (77.8)	17 (48.57)
Relapse	24 (34.3)	22 (30.1)	0 (0)	0 (0)	3 (33.3)	1 (11.1)	18 (51.4)
Unknown	1 (1.4)	1 (1.4)	0 (0)	0 (0)	0 (0)	1 (11.1)	0 (0)
**PANSS**							
Total score, median (IQR)	12 (6)	14 (8.8)	19 (6)	23 (12.5)**	11 (8.5)	12 (4.5)	13 (7)
Unknown, no.	0	0	1	0	0	0	0

The Kruskal-Wallis test with Dunn’s multiple comparisons test was used to identify significant differences of each class compared to Control for numerical variables (age, PANSS scores) while Fisher’s exact test was used for categorical variables. Ethnicity data was analysed by comparing White vs Combined Asian/Black/Mixed/Other ethnic groups in Ctrl, combined VGKC/GlyR Group, combine NMDAR/LGI1/CASPR2 Group, as applicable to Chi-square test/Fisher’s exact test. **p < 0.01.

*Ctrl* control, psychosis patients tested negative for the following neuronal cell surface antibodies. *Ab* antibody, psychosis patients tested positive for one of the following neuronal cell surface antibodies. *GlyR* glycine receptor. *VGKC* voltage-gated potassium channel complex. *CASPR2* contactin associated protein-like 2. *LGI1* leucine-rich glioma inactivated 1. *NMDAR* N-methyl-D-aspartate receptor. *PANSS* Positive and Negative Syndrome Scale. *IQR* Interquartile Range.

### Two distinct serum biochemical signatures were detected by unsupervised analysis, which correspond to VGKC antibody and GlyR antibody positivity

**Fig. 1:**
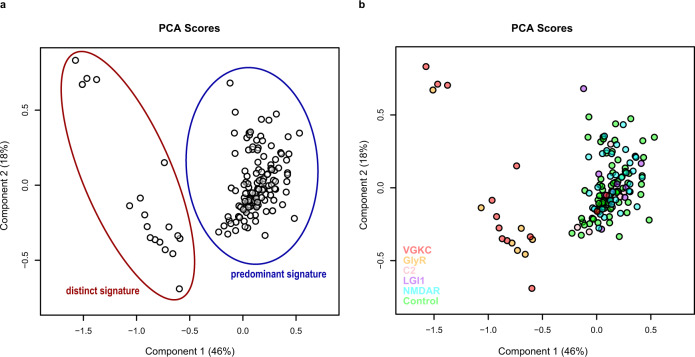
PCA scores plots of NMR serum metabolomics. **a** The NMR metabolite signature spontaneously separated the psychosis patient cohort into two groups: the ‘predominant biochemical signature group’ and the ‘distinct biochemical signature group’. **b** Psychosis patients who tested positive for serum antibodies against VGKC (red, *n* = 13) or GlyR (orange, *n* = 7) spontaneously separated from those who tested positive for C2 (pink, *n* = 9), LGI1 (purple, *n* = 9), or NMDAR (blue, *n* = 35) and antibody negative Control (green, *n* = 70) samples.

In an effort to identify novel psychosis subtypes within this cohort, we performed an untargeted analysis of a large panel of serum biochemicals (~100 NMR-detectable small molecules) associated with inflammation, the acute phase response, energy metabolism, amino acid degradation, and fatty acid oxidation. Unsupervised PCA (blinded to antibody status and all other demographic data) of the NMR metabolome alone, spontaneously separated the cohort in to two distinct groups of *n* = 18 and *n* = 125 (Fig. 1a). This suggests, that 18 of the patients in the cohort have distinct serum biochemical signatures when compared to all other samples. When demographic data was superimposed on to the unsupervised scores plot no association between this biochemically distinct group and age, gender, ethnicity, episode type, or disease duration was observed (Supplementary Fig. 2). However, a clear association with antibody status was observed (Fig. 1b). All antibody negative, NMDAR, LGI1 and CASPR2 antibody patients were found to fall with the predominant biochemical signature group while all but two VGKC antibody positive patients (85%, *n* = 11) and all GlyR antibody positive patients (100%, *n* = 7) fell within the ‘distinct biochemical signature’ group.

This suggests that the serum biochemical signature of NMDAR, LGI1, and CASPR2 antibody psychosis patients is indistinguishable from antibody negative psychosis patients. In contrast, VGKC and GlyR antibody psychosis patients have a shared biochemical signature which is distinct.

### Significant metabolic imbalances including decreases in serum lipoproteins along with increased amino acid concentrations were observed in VGKC and GlyR antibody positive psychosis patients

To further understand the biochemical perturbations associated with sub-groups identified above, the cohort was split in to three groups 1. A VGKC and GlyR antibody combined group (VGKC/GlyR, *n* = 20), 2. a group consisting of patients with NMDAR, LGI1, or CASPR2 antibodies (NMDAR/LGI1/CASPR2, *n* = 53), and 3. a control group of patients negative for all antibodies tested (Control, *n* = 70). Supervised OPLS-DA analyses was performed to build predictive models and tested on independent data (data that was not used to train the model).

**Fig. 2:**
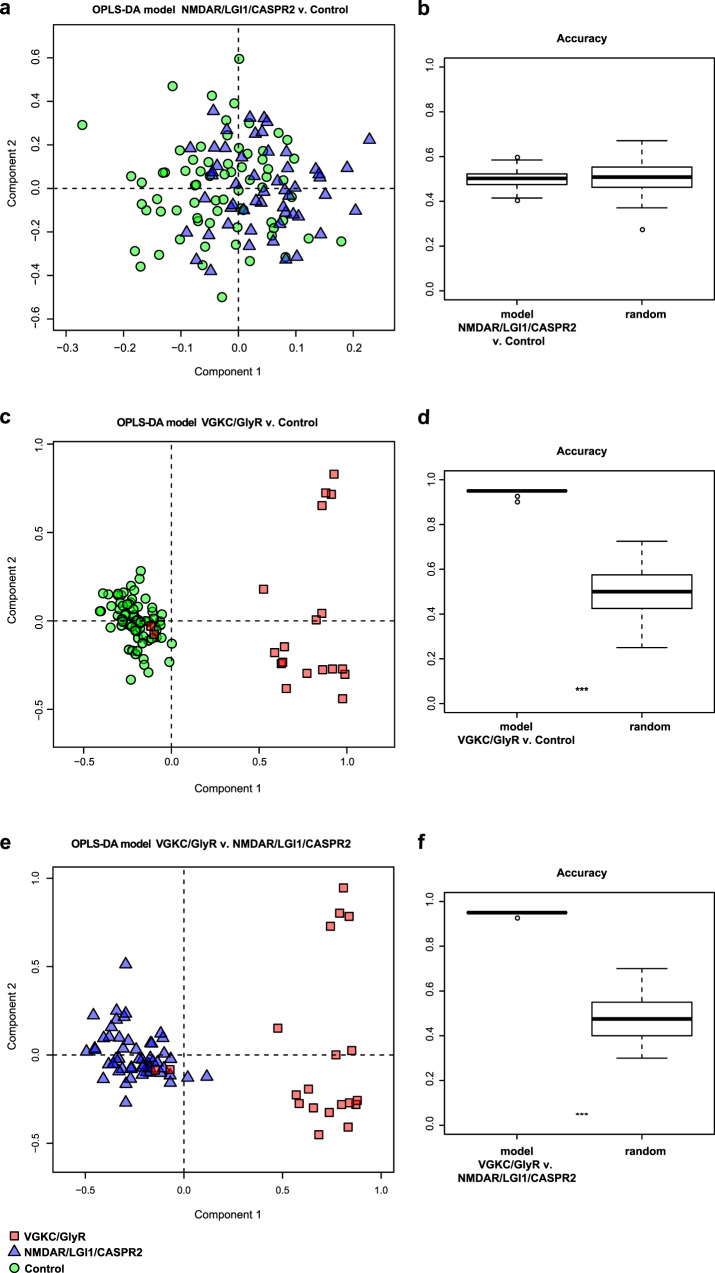
OPLS-DA models discriminating VGKC/GlyR (red square, *n* = 20) samples from NMDAR/LGI1/CASPR2 (blue triangle, *n* = 53) samples and Control (green circle, *n* = 70) samples using NMR serum metabolomic data. **a**, **c**, **e** OPLS-DA scores plots of NMDAR/LGI1/CASPR2 v. Control, VGKC&GlyR v. Control, VGKC/GlyR v. NMDAR/LGI1/CASPR2. **b**, **d**, **f** Predictive accuracy of the ensemble of the OPLS-DA models compared with that of the randomly permutated null distribution. Kolmogorov-Smirnov test. ***p < 0.001.

No separation was observed between the control and NMDAR/LGI1/CASPR2 antibody groups in the OPLS-DA scores plot (Fig. 2a) and the performance of the model, on independent test data, was not significantly better than that expected by random chance (Fig. 2b, *p* value > 0.05, Kolmogorov-Smirnov test) confirming there are no detectable differences in biochemical signature between these groups in this cohort. In contrast, OPLS-DA was able to predict which patients, in the test data, belonged to the VGKC/GlyR group with 94.78 ± 0.80% accuracy, 99.76 ± 1.21% sensitivity, and 92.75 ± 2.21% specificity (*p* values all <0.001, Kolmogorov-Smirnov) relative to controls (Fig. 2c, d) and 94.75 ± 0.75% accuracy, 99.60 ± 1.39% sensitivity, and 93.11 ± 2.12% specificity (*p* values all <0.001, Kolmogorov-Smirnov) relative to NMDAR/LGI1/CASPR2 positive patients (Fig. 2e, f).

**Fig. 3:**
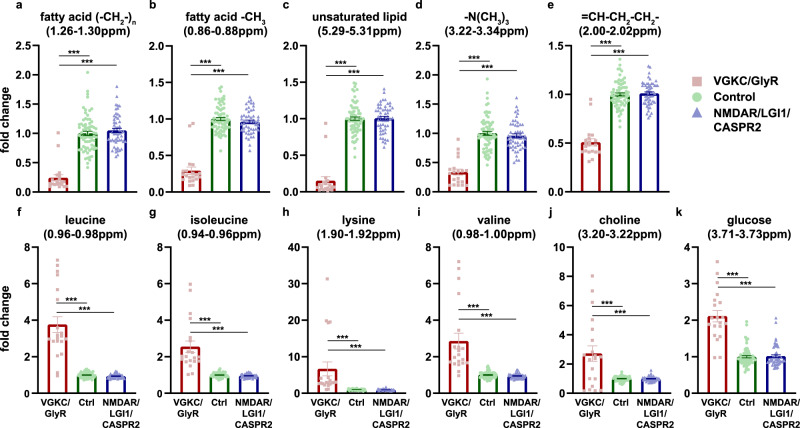
Levels of discriminatory serum metabolites selected by the OPLS-DA models in VGKC/GlyR (red, *n* = 20), control (green, *n* = 70), and NMDAR/LGI1/CASPR2(blue, *n* = 53) groups. **a**–**e** Decreased levels of several fatty acid resonances within serum lipoproteins (−CH3, (−CH2-)n, -N(CH3)3, unsaturated fatty acid, =CH-CH2-CH2−) in the VGKC/GlyR antibody group. **f**–**k** Increased levels of several amino acids (leucine, isoleucine, lysine, and valine), choline and glucose in the VGKC/GlyR antibody group. Error bars: ±SEM. One-way ANOVA with Tukey’s post-hoc corrections. ***p  <  0.001.

The metabolites perturbed in the VGKC and GlyR antibody psychosis patients were identified by inspection of the variable importance of projection (VIP) scores of the significant OPLS-DA models described above. Significant decreases were observed in several fatty acid resonances within serum lipoproteins (−CH_3_, (−CH_2_-)_n_, -N(CH_3_)_3_, unsaturated fatty acid, =CH-CH_2_-CH_2_−) while several amino acids (leucine, isoleucine, lysine, and valine) were increased in the VGKC and GlyR antibody patients (Fig. 3 and Supplementary Table 1, *p* values all <0.001, one-way ANOVA with Tukey’s post hoc test).

### Patients with elevated serum VGKC antibody or GlyR antibody have significantly higher PANSS ratings

To determine whether the metabolism disturbances identified in the VGKC and GlyR antibody patients were associated with a distinct clinical phenotype, we investigated the PANSS scores across these groups by comparing the median scores between groups. VGKC and GlyR antibody patients had significantly higher PANSS totals than either the antibody negative or NMDAR, LGI1, CASPR2 antibody patients (both *p* values < 0.001, Kruskal-Wallis test with Dunn’s multiple comparisons test) (Table 2). In exploring the subscale scores, the VGKC/GlyR antibody had higher positive symptoms, negative symptoms, and general symptoms than the control patient groups (*p* values < 0.05, <0.05, and <0.001, respectively, Kruskal-Wallis test with Dunn’s multiple comparisons test) (Table 2). In order to explore the possibility that the biochemical signature identified in the VGKC/GlyR group was a marker of severity of illness, we undertook a separate OPLS-DA model of high PANSS v low PANSS (independent of antibody status). This resulted in lower accuracy, sensitivity, and specificity of model, indicating that this metabolomic profile was not just a marker of illness severity (−30.82%, −28.91%, and −26.51% respectively).

**Table 2:** Comparison of PANSS scores of patients with VGKC/GlyR antibody, NMDAR/LGI1/CASPR2 antibody test, and antibody negative control groups.

	Ctrl	VGKC/GlyR	NMDAR/LGI1/CASPR2
**Number of patients**	70	19	53
**Age (years), Median (IQR)**	27 (15)	23 (8)	28 (15)
**Gender, Female, no. (%)**	31 (44.3)	8 (42.1)	21 (39.6)
**PANSS, Median (IQR)**			
Positive symptom score	6 (2.3)	9 (5)*	7 (3)
Negative symptom score	3 (3)	6 (6)*	3 (3)
General symptom score	2 (0)	5 (5)***	2 (0)^†††^
Total score	12 (6)	19 (8)***	12 (6)^†††^

The PANSS score for one VGKC/GlyR patient was not available and so was excluded from this table.

The Kruskal-Wallis test with Dunn’s multiple comparisons test was used to identify significant differences of each class for numerical variables (age, PANSS scores) while Fisher’s exact test was used for categorical variables. *Indicates significant differences of each class compared to Ctrl. ^†^indicates significant differences of each class compared to VGKC/GlyR. *p < 0.05, ***p < 0.001. ^†††^*p* < 0.001.

*Ctrl* control, psychosis patients tested negative for the following neuronal cell surface antibodies. *VGKC/GlyR* voltage-gated potassium channel complex & glycine receptor; psychosis patients tested positive for either antigen. *CASPR2* contactin-associated protein-like 2; LGI1 leucine-rich glioma inactivated 1, *NMDAR* N-methyl-D-aspartate receptor. *NMDAR/LGI1/CASPR2* psychosis patients tested positive for CASPR2/LGI1/NMDAR. *PANSS* Positive and Negative Syndrome Scale. *IQR* Interquartile Range.

## Discussion

Unsupervised and untargeted metabolomics analysis identified a biochemical signature of VGKC and GlyR antibody positive psychosis which consisted of decreased fatty acid lipoprotein levels along with increased leucine, isoleucine, valine, lysine, free choline, and glucose concentrations.

Serum lipoproteins are increasingly recognised to play a role in the immune system and active inflammation is commonly associated with decreased small lipoprotein particles such as high-density lipoproteins (HDL) and low density lipoproteins (LDL) coupled with an increase in triglyceride concentration and the larger, very low-density lipoprotein (VLDL) particles [38]. In addition, pro-inflammatory cytokines have been shown to induce dyslipidaemia [39] and lead to a reduction in HDL cholesterol [40]. Finally, increasing LDL cholesterol concentration and improving HDL cholesterol efflux have been shown to improve C-reactive protein (CRP) associated inflammation in diseases such as rheumatoid arthritis [41, 42]. Thus, the decrease in fatty acid lipoprotein signatures identified here are consistent with increased inflammation in the VGKC and GlyR antibody-positive groups.

Elevated plasma concentrations of branched chain amino acids (BCAA) and lysine have been previously reported in people with schizophrenia [43] and elevated BCAA inhibit transport of dopamine and serotonin precursors (tyrosine and tryptophan, respectively) into the brain, which can lead to anxiety and mood disorders [44, 45] Elevated BCAA may also lead to insulin resistance [46, 47] which is associated with first-episode psychosis [48]. The significant increase in glucose concentration observed here supports this and, along with the increased BCAA levels identified, may be associated with the increased PANSS scores observed in the VGKC and GlyR antibody positive patients.

Taken together, the metabolomics analysis suggests a distinct metabolomic phenotype in VGKC and GlyR antibody positive patients which is associated with increased PANSS scores at presentation, increased inflammation, and potentially decreased neurotransmitter precursors and insulin resistance. Future work, on a larger cohort, will investigate these pathways in more detail.

Our finding of a distinct metabolomic and clinical phenotype associated with VGKC and GlyR antibodies was somewhat against our original hypothesis. The neuronal cell surface antibodies with the strongest evidence for pathogenicity are the NMDAR, LGI1, and CASPR2 antibodies, with substantial case report evidence that they have a direct effect on neuronal function and cause the expression of neuropsychiatric illness. We, therefore, hypothesized that these antibodies would be particularly associated with biochemical markers of inflammation.

However, in spite of the accepted pathogenicity of these antibodies it has also been recognized, paradoxically, that classical measures of neuroinflammation may also be absent in these patients, whether in MRI scans, or in serum or CSF measures of inflammation [49, 50]. The absence of a distinctive metabolomic profile in those with NMDAR/LGI1/CASPR2 serum antibodies in psychosis does not necessarily indicate that these antibodies are not having an effect in the brain in these patients.

VGKC and GlyR antibodies, by contrast do not have such a strong literature to support their direct pathogenicity in neuropsychiatric disorders. A particularly influential paper, reviewing the presence of VGKC antibodies in the absence of LGI1 or CASPR2 antibodies in a clinical cohort at the Mayo clinic, found that the presence of the antibodies was not associated with an immunotherapy responsive illness [51, 52]. However, this clinical sample was of largely older people with a range of other degenerative diagnoses, and not comparable to a younger cohort of people without comorbidity. It is possible therefore, in these cases that the VGKC antibodies were a secondary, non-specific marker of neurodegeneration.

A further clinical study suggests that non-LGI1, non-CASPR2 VGKC antibodies are a non-specific marker of an inflammatory brain condition in children. A series of patients were tested for antibodies, and separately rated according to clinical and paraclinical measures into having a likely inflammatory brain condition, or not. Of 39 patients with these VGKC antibodies, 30 were considered clinically to have an inflammatory condition. The likelihood of an inflammatory condition was raised with a higher titre of the antibody [25]. There are further case reports of those with atypical dementia or pain syndromes and VGKC antibodies that are responsive to immunotherapy, suggesting that these antibodies may indeed be a marker of an inflammatory condition [53]. Further work is now required to investigate the biological function and relevance of titre level of these antibodies in psychosis.

GlyR is a glycine-gated chloride ion channel typically expressed on the surface of motor neurons in the brainstem and spinal cord, regulating neuronal excitability. GlyR is also found in human hippocampus, but its role here is less clear [54]. GlyR antibodies were first described in progressive encephalopathy with rigidity and myoclonus (PERM) [55], and not previously described in association with psychiatric presentations [56].

Taken together, it is likely therefore that these antibodies are acting as non-specific markers of a possible inflammatory aetiology in these patients, rather than indicating a more specific VGKC or GlyR pathology.

The association between the GlyR and VGKC antibodies and higher PANSS ratings overall indicates patients with these antibodies are less responsive to antipsychotic medication. The finding of greater negative symptoms in this group is in keeping with the notion that negative symptoms are the result of neuroinflammation, with inflammatory stimuli decreasing neural activity in the ventral striatum, decreasing connectivity in reward pathways, and resulting in a lack of motivation in patients [34].

There are limitations to this study. As this was a pilot study and a number of different antibodies were included, the numbers in each group were small. There was no significant difference in any of the potential confounders investigated (age, gender, ethnicity, episode type). Nonetheless, we investigated the effect of each potential confounder on the multivariate model and further confirmed that neither age, gender, ethnicity, nor episode type were contributing factors to the models (Supplementary Fig. 2).

Prescribed medication was not recorded in participants recruited after 2016 and it is possible that this could affect the metabolomic profile seen. However, in the samples collected prior to 2017 almost all patients were prescribed atypical antipsychotics, in keeping with usual clinical practice in UK. It is a reasonable assumption that similarly the later patients were also all prescribed an atypical antipsychotic. There would be no reason why those with antibodies would systematically be given a different medication, although we cannot prove this.

The samples were all collected in the same brand of serum separator tube that was supplied by the study team, and the samples were assessed by the same researcher for the presence of antibodies, reducing chance of any systematic bias. The samples were sent at room temperature to the study team. This led to a delay of a day or two before the samples were processed. This delay likely led to a degradation of samples, and a consequent lack of sensitivity to detect some metabolites. We did not measure motor symptoms in the patients to know whether there were any correlates with the GlyR antibodies. Finally, we did not include any other psychiatric or neurological groups, and therefore are unable to say whether this pattern is distinct to psychosis, or not.

These limitations do not detract from the main finding of a biochemically distinct subgroup of patients with first episode psychosis that is associated with higher PANSS scores and VGKC and GyR antibody positivity, which indicates a potential inflammatory aetiology for a proportion of patients, and gives the potential for different treatment approaches for these patients. Further work is now required to validate the biomarkers identified in a larger, prospective cohort and assess the performance of our multivariate diagnostic model to other inflammatory diseases of the central nervous system and routinely used inflammatory markers.

In conclusion we have demonstrated a distinct biochemical profile of a subgroup of patients with acute psychosis who have a more severe illness. This is particularly exciting, because it is these patients, resistant to current antipsychotic medication, that are in particular need of new therapeutic approaches. If confirmed, these findings could therefore lead to the trial of novel targeted treatments on the basis of individuals’ metabolomic profile.

## Supplementary information

Detailed statistical methods of validating OPLS-DA models

## Data Availability

Anonymized data and code will be shared by request from any qualified investigator.
