# Supplementary material for: The serum metabolomic profile of a distinct, inflammatory subtype of acute psychosis
Source: Mol Psychiatry. 2022 Sep 21;27(11):4722–30. doi: 10.1038/s41380-022-01784-4 (PMC7613906; doi:10.1038/s41380-022-01784-4)
Supplement: Supplementary file 1 — Detailed statistical methods of validating OPLS-DA models [file 41380_2022_1784_MOESM1_ESM.docx]

**The serum metabolomic profile of a distinct, inflammatory subtype of acute psychosis.**

Belinda Lennox DM^1^*, Wenzheng Xiong BSc^2,3^*, Patrick Waters PhD^4^, Alasdair Coles PhD^5^, Peter Jones PhD^6^, Tianrong Yeo MD PhD ^2,7,8^, Jeanne Tan May May MD PhD^7,8^, Ksenija Yeeles PhD^1^, Daniel Anthony PhD^2^, Fay Probert PhD^3^

1. Department of Psychiatry, University of Oxford and Oxford Health NHS Foundation Trust
2. Department of Pharmacology, University of Oxford
3. Department of Chemistry, University of Oxford
4. Nuffield Department of Clinical Neurosciences, University of Oxford
5. Department of Clinical Neurosciences, University of Cambridge
6. Department of Psychiatry, University of Cambridge
7. Department of Neurology, National Neuroscience Institute, Singapore.
8. Duke-NUS Medical School, Singapore

Corresponding author: Belinda Lennox, Department of Psychiatry, Warneford Hospital, Headington Oxford OX3 7JX.

* joint first authorship

**Corresponding author**: Belinda Lennox (belinda.lennox@psych.ox.ac.uk)

**Keywords:** antibody mediated, psychosis, metabolomics, inflammation

**Tables/Figures (max 5)**

**Word count excluding (abstract, refs) max. 3500**

**Supplementary Information**

**Detailed statistical methods of validating OPLS-DA models**

OPLS-DA is a supervised modelling method analysing pre-defined groups. It is a powerful tool to investigate discriminatory variables driving the separation of two groups. However, it has an innate tendency to overfit the data, especially when provided high-dimensional spectral data (Worley & Powers, 2016). Without rigorous validation, the resulting models would be false positive and unreliable. In this study, OPLS-DA models were validated using a 10-fold external cross-validation with repletion and permutation testing (Supplementary Figure 1).

The first step was to correct for unequal class sizes by randomly selecting samples from the larger class to match the number of samples in the smaller class. Then the data was randomly shuffled and split into a training set (90% of data) and a test set (10% of data). The training set was then used to build the OPLS-DA model optimised by 7-fold internal cross-validation as the default settings in the ropls package (v1.26.4; Thévenot *et al*., 2015). After the OPLS-DA model was built, the, previously unseen, test set was then employed to assess the predictive ability of the model by calculating the accuracy, sensitivity and specificity. The process of splitting the dataset was repeated 10 times so that each sample appears in a test set exactly once. And the entire process, which started from randomly selecting samples to 10-fold external cross-validation, were repeated a further 100 times and altogether to produce 1000 models in total.

Cross-validation described above assessed how well the model performs on an independent data, while the permutation test mentioned below checked whether the model performed significantly better than random chance (the null distribution). A random permutation using random class assignment was used. The classes of the data set were randomly permuted and then OPLS-DA models were built with 10-fold cross-validation to produce a null distribution. Only if the accuracy of the true models is significantly better (Kolmogorov-Smirnov test) than that expected by random chance (~50%) is the model considered to be significant. Then the separation observed between classes is confirmed and it is valid to interrogate metabolites driving the separation and to use the data for prediction of additional samples.

Supplementary Table 1. Discriminatory metabolites selected from the OPLS-DA models of VGKC/GlyR vs Control and OPLS-DA models of VGKC/GlyR vs NMDAR/LGI1/CASPR2

| Discriminatory metabolites | Chemical shift of spectral bins (ppm) | VGKC/GlyR : Control (*p* value) | NMDAR/LGI1/CASPR2 : Control | VIP score (VGKC/GlyR vs Control) | VIP score (VGKC/GlyR vs NMDAR/LGI1/CASPR2) |
| --- | --- | --- | --- | --- | --- |
| fatty acid (-CH_2_-)_n_ CM/VLDL | [1.26, 1.30] | 0.24 (<0.0001) | 1.05 | 3.41 | 3.52 |
| fatty acid -CH_3_ HDL/LDL | [0.86, 0.88] | 0.29 (<0.0001) | 0.96 | 3.24 | 3.07 |
| leucine | [0.96, 0.98] | 3.76 (<0.0001) | 0.94 | 3.00 | 2.99 |
| choline | [3.20, 3.22] | 2.72 (<0.0001) | 0.99 | 2.83 | 2.84 |
| -N(CH_3_)_3_ | [3.22, 3.24] | 0.34 (<0.0001) | 0.96 | 2.48 | 2.44 |
| isoleucine | [0.94, 0.96] | 2.55 (<0.0001) | 0.95 | 2.04 | 2.05 |
| lysine | [1.90, 1.92] | 6.68 (<0.0001) | 0.97 | 1.98 | 1.96 |
| unsaturated lipid | [5.29, 5.31] | 0.15 (<0.0001) | 1.01 | 1.90 | 1.89 |
| valine | [0.98, 1.00] | 2.86 (<0.0001) | 0.95 | 1.75 | 1.76 |
| =CH-CH_2_-CH_2_- | [2.00, 2.02] | 0.51 (<0.0001) | 1.01 | 1.70 | 1.69 |
| glucose | [3.71, 3.73] | 2.11 (<0.0001) | 1.01 | 0.65 | 0.44 |

The table shows fold changes of levels of metabolites selected from the OPLS-DA models and glucose. Metabolites shown above (except glucose) had a VIP score ≥1.7 (in the model of VGKC/GlyR vs Control) or ≥1.69 (in the model of VGKC/GlyR vs NMDAR/LGI1/CASPR2). The cut-off of VIP scores was decided based on the inflection point of the curve of VIP scores. The discriminatory metabolites in both models were identical, indicating they were metabolic signatures specific to the VGKC/GlyR group. *p* values were calculated using one-way ANOVA with Tukey’s post-hoc corrections.

CM: chylomicron. VLDL: very low-density lipoprotein. HDL: high density lipoprotein. LDL: low density lipoprotein. VIP: variable importance in projection. OPLS-DA: orthogonal partial-least square discriminant analysis.

Supplementary Figure 1. Schematic representation of cross-validation and permutation strategy to validate OPLS-DA models

Supplementary Figure 2. PCA scores plot of NMR serum metabolomics. The spontaneous separation of NMR metabolomics was not associated with age (a), gender (b), ethnicity (c), episode type (d), and disease duration (e).
